# Optimization and prospective evaluation of sensitive real-time PCR assays with an internal control for the diagnosis of melioidosis in Thailand

**DOI:** 10.1128/spectrum.01039-23

**Published:** 2023-10-11

**Authors:** Chawitar Noparatvarakorn, Wallop Jakkul, Rathanin Seng, Sarunporn Tandhavanant, Orawan Ottiwet, Rachan Janon, Wadsamon Saikong, Narisara Chantratita

**Affiliations:** 1 Department of Microbiology and Immunology, Faculty of Tropical Medicine, Mahidol University, Bangkok, Thailand; 2 Department of Medical Technology and Clinical Pathology, Mukdahan Hospital, Mukdahan, Thailand; 3 Department of Medicine, Mukdahan Hospital, Mukdahan, Thailand; 4 Mahidol-Oxford Tropical Medicine Research Unit, Faculty of Tropical Medicine, Mahidol University, Bangkok, Thailand; Tainan Hospital, Ministry of Health and Welfare, Tainan, Taiwan

**Keywords:** *Burkholderia pseudomallei*, melioidosis, PCR, TTS1-*orf2*, BPSS0745, BPSS1187, BPSS1498, BPSS0087, BPSS1492, internal control, diagnosis

## Abstract

Melioidosis is a severe infectious disease caused by *Burkholderia pseudomallei*. Effective treatment of melioidosis requires rapid detection of *B. pseudomallei* infection. Previous studies demonstrated that real-time PCR for the diagnosis of melioidosis in blood samples was insensitive. We optimized the DNA extraction method and evaluated real-time PCRs with an internal control for the detection of *B. pseudomallei* in plasma samples and compared the performance of real-time PCRs based on six target genes. The lower limit of detection (LOD) of TTS1-*orf2*, BPSS0745, BPSS1187, and BPSS1498 real-time PCRs was ≤10 genome equivalent (GE)/reaction, while the LOD of BPSS0087 and BPSS1492 was 100 GE/reaction. In the initial phase, we evaluated four targets (TTS1-*orf2*, BPSS0745, BPSS1187, BPSS1498) for the diagnosis of melioidosis using 87 plasma samples from 38 bacteremic-melioidosis patients and 49 other infection patients at a hospital in Northeast Thailand. Among these, BPSS1187 exhibited the highest sensitivity of 76.3% and specificity of 100%. A second evaluation phase was conducted at two hospitals in Northeast Thailand to compare two real-time PCRs between BPSS1187 and widely used TTS1-*orf2* targets. A total of 705 clinical samples were collected from 421 suspected melioidosis cases; 77 of them were subsequently confirmed as melioidosis by culture using one or more specimens. The sensitivities of BPSS1187 and TTS1-*orf2* were 89.6% and 79.2%, respectively (*P* = 0.004), and the specificities were 96% and 99%, respectively. The sensitivities of these two assays in plasma samples were 84.6% and 67.7%, respectively (*P* = 0.007). Therefore, the BPSS1187 real-time PCR was sensitive and specific for early diagnosis of melioidosis.

**IMPORTANCE:**

Melioidosis is a serious infectious disease caused by *Burkholderia pseudomallei*, an environmental Gram-negative bacterium. Early detection of *B. pseudomallei* infection is crucial for successful antibiotic treatment and reducing mortality rates associated with melioidosis. Bacteria culture is currently used to identify *B. pseudomallei* in clinical samples, but the method is slow. Therefore, there is a need for more accurate and sensitive molecular-based diagnostic methods that can detect *B. pseudomallei* in all sample types, including samples from blood. We developed an optimal DNA extraction method for *B. pseudomallei* from plasma samples and used an internal control for real-time PCR. We evaluated six PCR target genes and identified the most effective target for the early detection of *B. pseudomallei* infection in patients. To prevent delays in the treatment of melioidosis that can lead to fatal outcomes, we recommend implementing this new approach for routine early detection of *B. pseudomallei* in clinical settings.

## INTRODUCTION

Melioidosis is a fatal infectious disease caused by environmental Gram-negative bacteria, *Burkholderia pseudomallei*. This bacterium is commonly found in soil and water in tropical and subtropical regions where the disease is endemic (1). Melioidosis is acquired through inhalation of particles of contaminated soil, inoculation, or ingestion of contaminated food and water. The clinical manifestations of melioidosis can vary from localized cutaneous manifestations to severe sepsis and death (2). The northeast region (NE) of Thailand and northern Australia have reported the highest number of clinical cases. In NE Thailand, approximately 2,000 cases per year with a mortality rate exceeding 30% have been reported (3, 4). Melioidosis is the third most common cause of death from infectious diseases after human immunodeficiency virus/acquired immunodeficiency syndrome and tuberculosis (2, 4). Melioidosis was estimated to affect 165,000 people globally per year by predictive modeling, which highlighted that melioidosis is underdiagnosed and underreported (5).

Patients with suspected melioidosis mostly present with a septicemic illness. Bacteremia and pneumonia occur in 40%–60% of all patients. Bacterial dissemination to internal organs such as the spleen, liver, kidneys, and prostate is common. Septic shock occurred in around 20% of all cases (2, 6). The lack of accurate and early diagnosis is a significant factor in misdiagnosis and often causes delays in commencing antibiotic therapy for melioidosis, thereby leading to a high mortality rate (7). The current gold standard for laboratory diagnosis is a bacterial culture from a clinical sample. The sensitivity of culture using mathematical modeling was estimated at only 60.2% (8). Bacteremia was detected in up to 73% of melioidosis cases; however, an automated blood culture system demonstrated slow detection, and only 62.5% of cases were detected within 24 h (9). These factors result in ineffective treatment for melioidosis and a poor outcome for the patients.

Several nonculture-based rapid diagnostic tools have been developed and evaluated. A point-of-care lateral flow assay using a monoclonal antibody for *B. pseudomallei* capsular polysaccharide was evaluated for antigen detection, but the sensitivity was only 40% in stored whole-blood samples (10). The detection of *B. pseudomallei* in the blood may be challenging due to the low bacterial concentration at a median concentration of 1.1 CFU/mL (11). Serological diagnosis is an inexpensive method; however, the interpretation of the result is difficult due to the persistence of antibodies and the presence of cross-reactivities among anti-bacterial antibodies, especially in areas with a high prevalence of melioidosis and other tropical diseases. An indirect hemagglutination assay (IHA) is also widely used for the serodiagnosis of melioidosis. The assay is based on the detection of antibodies against whole bacteria of *B. pseudomallei*. IHA demonstrates low specificity of 68%–72% in culture-confirmed melioidosis cases in Thailand, and 28% of the healthy population in this country were reported to be seropositive, showing high background antibody levels (12, 13). A rapid immunochromatography test (ICT) based on hemolysin co-regulated protein 1 (Hcp1) was developed to detect a specific IgG antibody. Hcp1-ICT was prospectively evaluated in Thai patients with melioidosis. Based on bacterial culture results, the sensitivity of Hcp1-ICT was 88.3%, the specificity for Thai healthy donors was 86.1%, and that for U.S. donors was 100% (14). Many studies have demonstrated that IgM is not sensitive and specific for the diagnosis of melioidosis (15 – 17). Therefore, there is a need for a sensitive and specific test that can be helpful for early detection of melioidosis before patients develop detectable antibodies.

A PCR-based assay can be a primary method for the early detection of melioidosis. The most widely used target of PCR for melioidosis is a type III secretion system gene cluster 1, TTS1-*orf2* (18, 19). Recently, TTS1-*orf2* was prospectively evaluated with Hcp1-ICT in Thailand using clinical specimens collected from patients on the first day of admission. The TTS1-*orf2* real-time PCR demonstrated 78.2% sensitivity in all clinical samples, but only 34.6% sensitivity in blood samples of culture-confirmed melioidosis cases (20). The TTS1-*orf2* real-time PCR is highly specific, showing 100% specificity for all clinical samples of patients with other infections. When combined with Hcp1-ICT, the sensitivity increased to 98.2% (20). The selection of a PCR target is critical for the early diagnosis of melioidosis. Other genes, including BPSS0745, BPSS0087, BPSS1187, BPSS1498 (*hcp1*), and BPSS1492 (*bimA*), are promising targets for *B. pseudomallei* detection (21 – 24). However, the best target and whether multiple gene targets are required to obtain accurate results for the routine clinical diagnosis of melioidosis are unknown.

The accurate result of real-time PCR requires an appropriate reaction structure. This study aimed to optimize the DNA extraction conditions and evaluate sensitive and specific real-time PCR assays for the clinical diagnosis of melioidosis. We developed the optimal conditions for DNA extraction from *B. pseudomallei* using a new internal control construct and compared the performance of real-time PCRs that amplified six target genes, including TTS1-*orf2*, BPSS0745, BPSS0087, BPSS1187, BPSS1498 (*hcp1*), and BPSS1492 (*bimA*). Real-time PCR conditions for five genes, including TTS1-*orf2* (25), BPSS0745 (21), BPSS0087 (21), BPSS1187 (22), and BPSS1492 (*bimA*) (26), were previously developed (21, 22, 25, 26). A real-time PCR condition for BPSS1498 (*hcp1*) was developed in this study. The four most promising targets were clinically evaluated for the diagnosis of melioidosis in two hospitals in Northeast Thailand.

## MATERIALS AND METHODS

### Primer design and *in silico* identification of *B. pseudomallei*-specific targets

Real-time PCR assays were initially optimized using DNA from *B. pseudomallei* K96243 to amplify a region of six target genes as follows: TTS1-*orf2* (25), BPSS0745 (21), BPSS0087 (21), BPSS1187 (22), BPSS1498 (*hcp1*), and BPSS1492 (*bimA*) (26). These genes were described previously as potential PCR targets for *B. pseudomallei* identification. In this study, we followed the PCR primers and probes for five PCR targets developed from previous studies: TTS1-*orf2* (25), BPSS0745 (21), BPSS0087 (21), BPSS1187 (22), and BPSS1492 (*bimA*) (26). The PCR primers and probe for BPSS1498 (*hcp1*) were developed in this study. *In silico* analysis was performed to test the specificity of primers and probes (Fig. S1A). Primer-BLAST (https://www.ncbi.nlm.nih.gov/tools/primer-blast/) and Nucleotide Basic Local Alignment Search Tool (BLASTn, http://www.ncbi.nlm.nih.gov/BLAST/) with the default option were used to obtain the PCR target sequences for each target. This was achieved by interrogating our genome data for 1,294 *B. pseudomallei* clinical isolates from patients in Northeast Thailand (Table S1) (Seng et al., manuscript submitted). The obtained PCR target sequences for each gene were aligned using MAFFT v.7 (27). All PCR target sequence alignments were subjected to CD-HIT-EST v.4.8.1 (28) with a 100% threshold to identify the variation of each target sequence and then clustered into different variance groups. The region for PCR targets was selected based on the conserved regions of *B. pseudomallei,* and these regions are not present in the genomes of other organisms.

### Optimization of DNA extraction

Optimization of DNA extraction was initially performed using *B. pseudomallei* K96243. DNA was extracted from *B. pseudomallei*-spiked phosphate-buffered saline (PBS) using a QIAamp DNA Mini Kit or QIAamp DNA Blood Midi Kit (Qiagen, Hilden, Germany) and assessed by observation of the cycle threshold (Ct) value of TTS1-*orf2* real-time PCR on a CFX96 Touch Real-Time PCR System (Bio-Rad, Hercules, CA, USA) (Fig. S1). *B. pseudomallei* K96243 was cultured in Luria-Bertani broth at 37°C with shaking at 200 rpm for 16–18 h and used to inoculate PBS. To determine the optimal sample volume, DNA extraction was performed with uncentrifuged 200 µL, 1 mL, and 2 mL of 10^5^ CFU/mL *B. pseudomallei* K96243-spiked PBS (Fig. S1B). DNA extraction was compared with that extracted from the pellet of *B. pseudomallei*-spiked PBS. To optimize bacterial pellet preparation, centrifugation for 10, 20, and 30 min was performed (at 17,000 × *g*) in triplicate with 2 mL of *B. pseudomallei*-spike PBS. The DNA elution volume was 140 µL for extraction with a QIAamp DNA Blood Midi Kit and 60 µL for a QIAamp DNA Mini Kit.

### Determination of limit of detection

The limit of detection (LOD) of real-time PCR was determined for specific primers for all six targets using SYBR Green I (Bio-Rad, Hercules, CA). This method was used because it allows for the detection of multiple targets in a cost-effective way. The target sequences included TTS1-*orf2* (25), BPSS0745 (21), BPSS1187 (22), BPSS1498, BPSS0087 (21), and BPSS1492 (26). DNA from *B. pseudomallei* K96243 was quantified by a NanoDrop Lite spectrophotometer (Thermo Fisher Scientific, Waltham, Massachusetts, USA). LOD is presented as the number of *B. pseudomallei* genome equivalent (GE) per PCR reaction. One GE was calculated at approximately 7.9 fg, based on the *B. pseudomallei* K96243 genome size of 7.25 Mb (29). The amount of DNA was converted to GE copies. Genomic DNA was serially diluted from 1 × 10^6^ to 1 GE/reaction. Nine replicates were run for the high DNA concentrations from 1 × 10^6^ to 1 × 10^3^ GE/reaction, and 12 replicates were performed for the low concentration (1 × 10^2^ to 1 GE/reaction). The LOD was defined as the concentration at which positive reaction results were observed in 95% or more of the replicated reactions.

### Real-time PCR

Real-time PCR using SYBR Green I was initially optimized and performed on *B. pseudomallei* K96243 DNA to determine the sensitivity (LOD) of the six targets. Primers were synthesized by Bionic, Korea (Table 1). A temperature gradient PCR was performed to select an optimal annealing temperature for each target. The total reaction volume was 11 µL, consisting of 1× iTaq SYBR Green Supermix (Bio-Rad), 0.2 µM of forward and reverse primers, and 1 µL of total genomic DNA. The remaining reaction volume was maintained by adding a volume of molecular biology-grade water (HyClone, Cytiva, USA). The thermal cycling conditions were 95°C for 5 min, followed by 45 cycles at 95°C for 15 s, and at 61°C for 30 s. PCR products were validated by melting curve analysis from 65°C to 95°C, with an increment of 0.5°C, with continuous fluorescence monitoring.

**TABLE 1:** Sequence of primers and probes for the detection of *B. pseudomallei* and PCR products

PCR assay (product size)	Primer or probe	Oligonucleotide sequence	Concentration (nM)	References
TTS1-*orf2* (115 bp)	BpTT4176 forward	CGTCTCTATACTGTCGAGCAATCG	500	Novak et al., 2006 (25)
	BpTT4290 reverse	CGTGCACACCGGTCAGTATC	500	Novak et al., 2006 (25)
	BpTT4208 probe	FAM-CCGGAATCTGGATCACCACCACTTTCC-BHQ1	200	Novak et al., 2006 (25)
BPSS0745 (132 bp)	BPSS0745-Afw	GCGAGCAAATCCGTCTCTCA	500	Göhler et al., 2017 (21)
	BPSS0745-Fsrev	ATGCCAGGGCACATGGCTA	500	Göhler et al., 2017 (21)
	BPSS0745 probe	FAM-ATCATTCAGGCGGGTGCCGT-BHQ1	200	Göhler et al., 2017 (21)
BPSS1187 (81 bp)	8653 forward	ATCGAATCAGGGCGTTCAAG	500	Supaprom et al., 2007 (22)
	8653 reverse	CATTCGGTGACGACACGACC	500	Supaprom et al., 2007 (22)
	8653 probe	FAM-CGCCGCAAGACGCCATCGTTCAT-BHQ1	300	Supaprom et al., 2007 (22)
BPSS1498 (*hcp1*) (157 bp)	BPSS1498 forward	CCTCGCCTTCAAGAACGACTAC	500	This study
	BPSS1498 reverse	CTCTTCCATCATCTCGCGCTTG	500	This study
	BPSS1498 probe	FAM-CAGATCGTCGCCGCAATTCCTGCAG-BHQ1	200	This study
BPSS0087 (88 bp)	BPSS0087-Lfw	GAATGCGTGCGCGAGCA	200	Göhler et al., 2017 (21)
	BPSS0087-Brev	CTCGGCCGGTCCGGAAT	200	Göhler et al., 2017 (21)
BPSS1492 (*bimA*) (122 bp)	bpss1492_F	CATCCGAAACGCCGTCAATC	200	Saiprom et al., 2020 (26)
	bpss1492_R	CGTCGCCCGGATTTTTCTTC	200	Saiprom et al., 2020 (26)
TTS1-Ava (115 bp)	BpTT4176 forward	CGTCTCTATACTGTCGAGCAATCG	500	Novak et al., 2006 (25)
	BpTT4290 reverse	CGTGCACACCGGTCAGTATC	500	Novak et al., 2006 (25)
	TTS1_Ava probe	HEX-CTCCGTTGAGTAGTTTGGGTCATCCGG-BHQ1	200	Noparatvarakorn et al., 2023 (20)

TaqMan real-time PCR assays targeting TTS1-*orf2*, BPSS0745, BPSS1187, and BPSS1498 were performed with a total volume of 11 µL using a 1× SensiFAST Probe No-ROX Kit (Bioline, Australia). The sensitivity was tested with *B. pseudomallei* K96243 and 31 representative isolates of all variant groups for each target (Table S2). The final concentrations of the primers and probes are shown in Table 1. Genomic DNA (4 µL) extracted from clinical samples was used. The thermal cycling conditions consisted of 95°C for 5 min, followed by 95°C for 15 s, and 58°C for 30 s, with 45 cycles and a ramp rate of 3.3°C/s. The primer and probe concentrations and PCR conditions were modified in our study to achieve the optimal performance of our real-time PCR platforms. We adhered to the Minimum Information for Publication of Quantitative Real-Time PCR Experiments guidelines (30) to ensure the accurate and transparent reporting of our experiments.

The positive control constituted 100 pg of *B. pseudomallei* K96243 DNA. Negative control was molecular biology-grade water. Real-time PCR with a CFX96 Touch Real-Time PCR System using Bio-Rad CFX Maestro software (version 4.1.2433.1219) (Bio-Rad) was used during optimization and initial clinical evaluation. In the second evaluation phase, the Quantstudio 1 Real-Time PCR system using QuantStudio Design & Analysis Software v1.5.2 (Applied Biosystems, USA) was used at Mukdahan Hospital, and a CFX96 Touch Real-Time PCR System (Bio-Rad) was used at Roi Et Hospital.

### Internal control

TTS1-Ava DNA (2.5 pg) was added to each clinical sample of the patients as an internal control for DNA extraction and real-time PCR at the second evaluation phase. The TTS1-Ava internal control was constructed by substituting TTS1-*orf2* DNA with double-stranded oligonucleotides of *Angiostrongylus vasorum* cytochrome *c* oxidase subunit I. This region was a target for specific probe hybridization on a 115-bp TTS1-*orf2* PCR product (25). The modified fragment was ligated into a pUC57 plasmid (Bionic, Korea), referred to as TTS1-Ava (20). The TTS1-Ava internal control was used in the interpretation of samples reported as having no target detected for *B. pseudomallei* by real-time qPCR assays. The acceptable range of a Ct value is less than 35.

### Testing of PCR inhibitor in plasma

Inhibitors in plasma can reduce PCR sensitivity. The potential presence of an inhibitor in plasma was examined by comparing the PCR sensitivity between *B. pseudomallei*-spiked PBS and *B. pseudomallei-*spiked plasma. The bacteria were resuspended in PBS or pooled plasma from healthy donors, and the optical density was adjusted at 600 nm to be 1 × 10^8^ CFU/mL. Equal volumes of plasma were pooled from each of the four healthy donors. The plasma samples were randomly selected for testing for the presence of inhibitors. The bacterial suspension was serially diluted with PBS or plasma from 10^6^ to 10^1^ CFU/mL. Blood samples were drawn from healthy donors into Vacutainer K2 EDTA tubes (BD Diagnostics, Franklin Lakes, NJ, USA). Plasma was collected by centrifugation of blood at 1,500 × *g* for 15 min. DNA was extracted from 2 mL of *B. pseudomallei-*spiked PBS or plasma with a final elution of 140 µL using a QIAamp DNA Blood Midi Kit as instructed by the manufacturer (Qiagen, Hilden, Germany). Each was performed in triplicate (six dilutions of spiked PBS and six dilutions of spiked plasma performed in triplicate, total = 36). Unspiked PBS or plasma was used as a negative control. The colony count was determined for each dilution on Columbia agar in triplicate. The plates were incubated at 37°C for 16 h.

### Clinical evaluation

A prospective clinical evaluation of real-time PCR was conducted in two phases at two hospitals in Northeast Thailand (Fig. S1C). The initial evaluation phase was conducted using plasma samples at Mukdahan Hospital between October 2019 and November 2020. Subsequently, the second evaluation phase was conducted using plasma and other specimen types at both Mukdahan and Roi Et Hospitals between April 2022 and September 2022. Patients with suspected melioidosis were identified during the daily ward rounds. The number of suspected cases for the initial and second evaluation phases was 87 and 421, respectively. Clinically suspected melioidosis cases were identified by clinicians at the hospitals with the following criteria: (i) sepsis defined as an infection with organ dysfunction in accordance with the Third International Consensus (Sepsis-3) guidelines for sepsis (31, 32) and (ii) patients without sepsis but with one of the following conditions: fever (>38°C) or low temperature (<36°C), and any of the following diseases: diabetes mellitus (underlying disease or first diagnosis based on the diagnostic criteria of the American Diabetes Association criteria) (33, 34), chronic kidney disease (35), or thalassemia. Exclusion criteria were admission to other hospitals with a total admission time >72 h, pregnancy, receiving palliative care, or incarceration.

### Clinical samples and bacterial culture

Clinical samples were collected from suspected melioidosis cases for culture and real-time PCR. Samples, including blood, pus, sputum, urine, and body fluids, were collected and processed at the study sites as previously described (20). A 5-mL blood sample was obtained in a Vacutainer K2 EDTA tube (BD Diagnostics) for real-time PCR testing, and 10-mL blood was obtained for standard blood culture from the microbiology laboratory of the hospitals. Blood culture at Mukdahan Hospital was performed using BacT/Alert 3D (bioMérieux, Marcy l’Étoile, France) or DL-Bt240 (Zhuhai DL Biotech, Guangdong, China) with 5 days of incubation and at Roi Et Hospital using BacT/Alert Virtuo (bioMérieux) with 7 days of incubation. Biochemical identification and antibiotic susceptibility testing were performed as described in the ASM’s *Clinical Microbiology Procedures Handbook* (36). Pus was obtained using a disposable sterile cell harvester (Jiangsu Jianyou Medical Technology, Jiangsu, China) and resuspended in 500 µL of sterile PBS. Sputum was collected and mixed with an equal volume of sterile 4% NaOH. Pus, sputum, urine, and body fluid samples obtained were cultured in standard media of the microbiology laboratory, e.g., blood agar and MacConkey agar, at 37°C for 2 days. Stored clinical samples at −80°C were used for DNA extraction in the initial evaluation, and freshly collected samples were used for the second phase evaluation.

Melioidosis cases were defined by positive *B. pseudomallei* culture in any clinical sample. Non-melioidosis patients were defined as those in whom other pathogenic microorganisms were detected by bacterial culture, as reported by the microbiology laboratories of Mukdahan Hospital or Roi Et Hospital. Patients with mixed infections were included in this study. Bacterial culture with susceptibility testing was identified and performed in all study hospitals using the standard operating procedures by the Department of Medical Science, Ministry of Public Health, Thailand, Clinical and Laboratory Standards Institute (CLSI guidelines), and Thailand medical technology standard by laboratory accreditation of the Medical Technology Council.

### DNA extraction from clinical samples

DNA was extracted from a pellet suspension of 2 mL of plasma and 10 mL of urine. Plasma and urine pellets were obtained by centrifugation at 17,000 × *g* for 10 min and 4,500 × *g* for 30 min, respectively. Pus, sputum, and body fluid were used for extracting DNA from 200 µL of uncentrifuged samples. The DNA elution volume was 60 µL for all the clinical samples.

### Statistical analysis

Statistical analyses were performed using Prism 8 Statistics (GraphPad Software Inc., La Jolla, CA, USA) and Stata version 14 (Stata Corp. LP, College Station, TX, USA). Continuous variables are presented as the mean and standard deviation (SD). Categorical variables are presented as percentages. A one-way analysis of variance was used to determine the reproducibility of DNA extraction conditions. Tukey’s multiple comparisons test was used to compare the Ct values of the paired groups. The sensitivity and specificity were calculated using bacterial culture results as a gold standard. The McNemar test was used to compare the sensitivity and specificity between tests.

## RESULTS

### *In silico* analysis of *B. pseudomallei*-specific targets

**FIG 1:**
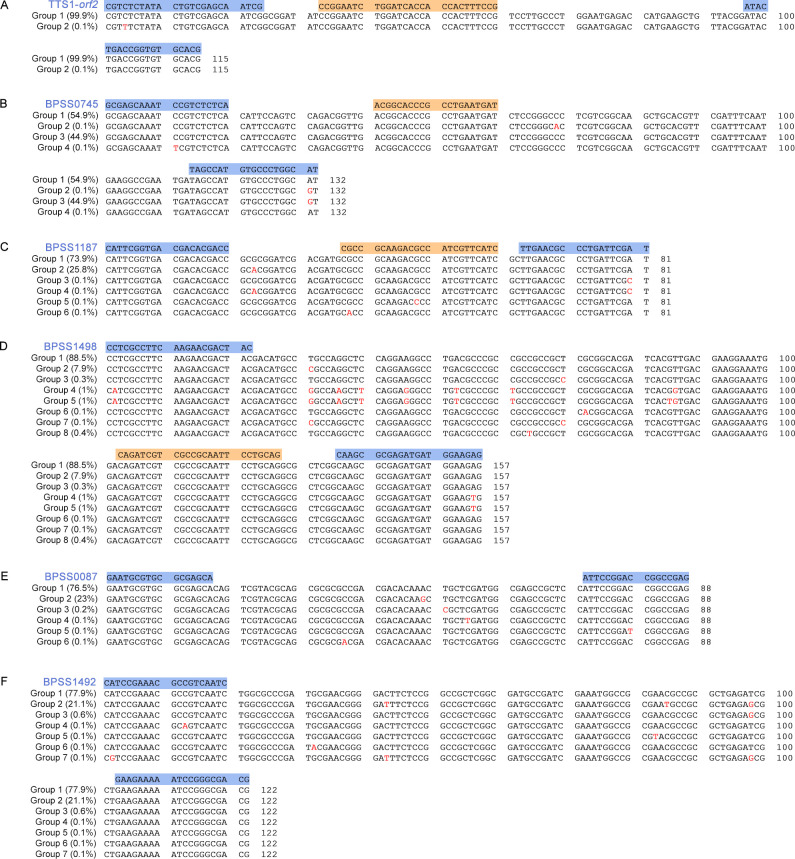
Names and locations of PCR target sequences. The single nucleotide polymorphisms (SNPs) of *B. pseudomallei* variants in relation to the reference strain K96243 and the percentage of each variant group are shown. Blue highlights the locations of primer binding. Orange highlights the location of probe binding. Red letters represent the locations of SNPs. (A) TTS1-*orf2*; (B) BPSS0745; (C) BPSS1187; (D) BPSS1498; (E) BPSS0087; (F) BPSS1492.

Six real-time PCRs have been optimized and evaluated to detect *B. pseudomallei* DNA rapidly. Genomic analysis of conserved and unique targets from 1,294 *B. pseudomallei* isolates revealed variation in the DNA sequences of PCR product regions. The number of variant groups from our genome data (Seng et al., manuscript submitted) and BLASTn of the National Center for Biotechnology Information (NCBI) database for TTS1-*orf2* (25), BPSS0745 (21), BPSS1187 (22), BPSS1498 (this study), BPSS0087 (21), and BPSS1492 (26) were 2, 4, 6, 8, 6, and 7, respectively (Fig. 1; Table S3). The Primer-BLAST analysis included 1,073 (TTS1-*orf2*), 994 (BPSS0745), 784 (BPSS1187), 2,403 (BPSS1498), 398 (BPSS0087), and 1,056 (BPSS1492) blast hits in the NCBI database (1 July 2021). *In silico* Primer-BLAST analysis of NCBI demonstrated that none of these targets were present in other organisms, with the exception of BPSS1498 in *B. mallei*.

### Sensitivity of real-time PCR

Initial SYBR Green I real-time PCR assays with 1 µL of DNA template were used to determine LOD. Of six targets, the LOD of four targets, including TTS1-*orf2,* BPSS0745, BPSS1187, and BPSS1498, was calculated to be 10 GE/reaction, and the LOD of two targets (BPSS0087 and BPSS1492) was one order of magnitude higher (100 GE/reaction) (Table S4). Based on the higher sensitivity, the four targets, TTS1-*orf2,* BPSS0745, BPSS1187, and BPSS1498, were selected for TaqMan real-time PCR optimization (Fig. S1D).

TaqMan real-time PCR assays were optimized for concentrations of primers and probes, time, and temperature and tested for sensitivity with genomic DNA from 31 representative *B. pseudomallei* isolates of different variant groups (Fig. S1; Table S3). Details of the isolates, variant groups, and Ct values are shown in Table S2. All *B. pseudomallei* isolates, including variants, were tested to be positive with similar Ct values by all real-time PCR assays (Table S2).

### Optimization of DNA extraction methods using *B. pseudomallei-*spiked PBS

**FIG 2:**
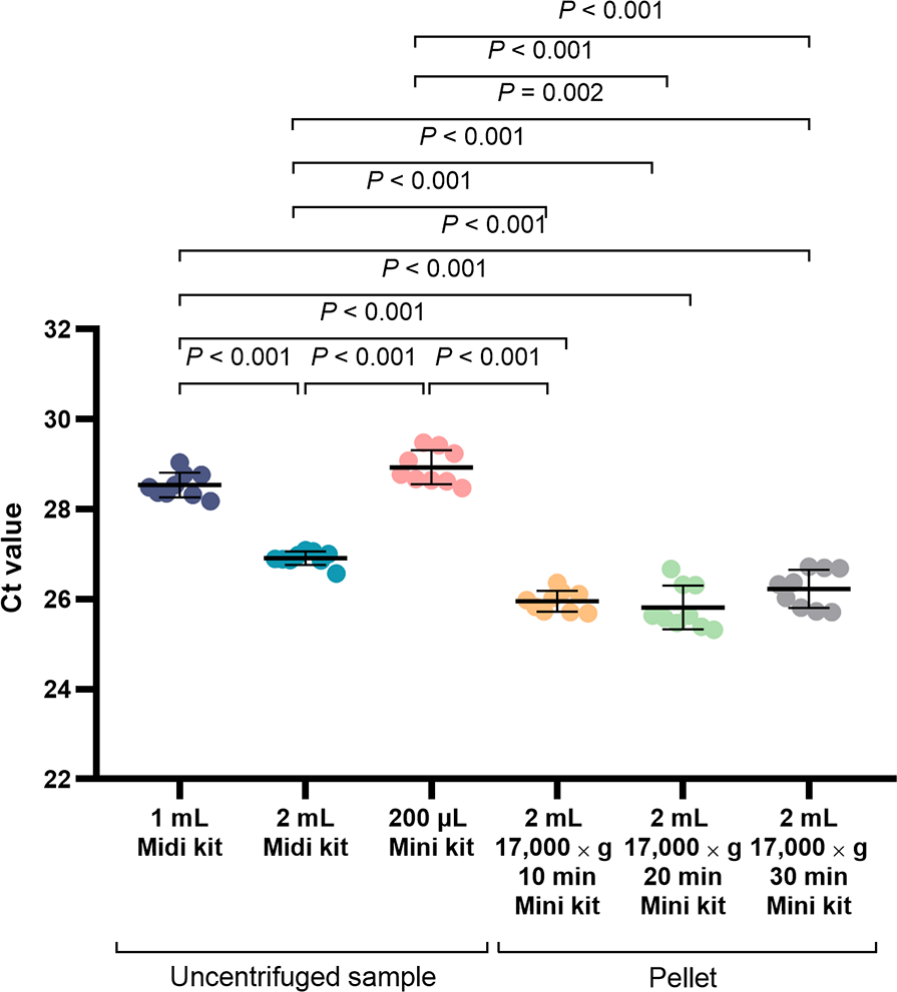
Comparison of different DNA extraction conditions for detecting *B. pseudomallei* K96243-spiked PBS. TTS1-*orf2*-PCR was performed using 1 × 10^5^ CFU/mL, with three independent extractions, and each was performed in triplicate (*N* = 9 per condition). The middle lines represent the mean cycle threshold values of the TTS1-*orf2*-PCR, with error bars representing standard deviations.

Six DNA extraction protocols using the QIAamp DNA Blood Midi Kit and QIAamp DNA Mini Kit were performed using 10^5^ CFU/mL *B. pseudomallei* K96243-spiked PBS samples (Fig. 2). To determine the optimal sample volume, DNA was extracted from 200 µL, 1 mL, and 2 mL of uncentrifuged samples. PCR reactions using 2 mL samples showed the highest sensitivity (lowest Ct value). To increase the highest DNA concentration, DNA was extracted from the pellet prepared by centrifugation of 2 mL of *B. pseudomallei* K96243-spiked PBS samples for 10, 20, and 30 min (Fig. 2). DNA extraction was performed in triplicate for each sample. The TTS1-*orf2*-PCR assay was performed in triplicate for each DNA extraction method to detect *B. pseudomallei* DNA. A total of 18 assays for each sample were performed (6 assays per sample per QIAamp DNA Blood Midi Kit and 12 assays per sample per QIAamp DNA Mini Kit). Reactions using pellets from three centrifugation times showed no significant difference. The means of Ct values and SD at 10, 20, and 30 centrifugation times were 25.96 (0.23), 25.82 (0.49), and 26.23 (0.42), respectively (Fig. 2).

The extraction method for blood samples was derived from the above optimization (Fig. 2) and standardized throughout the remaining parts of this study using the QIAamp DNA Mini Kit to extract DNA from bacterial pellets. The pellet was prepared from 2 mL of plasma sample by centrifugation at 17,000 × *g* for 10 min (Fig. 2). Two hundred microliters of pellet suspension was used for DNA extraction with an elution volume of 60 µL.

### Testing of potential PCR inhibitors in plasma

Human plasma samples may contain inhibitors for real-time PCR. To observe the effect of potential inhibitors in the plasma samples, we compared the PCR results between *B. pseudomallei*-spiked PBS and *B. pseudomallei*-spiked plasma. *B. pseudomallei* was spiked into sterile PBS and into pooled plasma samples of healthy individuals to obtain the final concentrations of 1 × 10^1^ to 1 × 10^6^ CFU/mL. The TTS1-*orf2* real-time PCR was performed with 2 mL of *B. pseudomallei*-spiked plasma sample and 2 mL of *B. pseudomallei*-spiked PBS (30). There was no significant difference in the Ct value for the detection of *B. pseudomallei* between spiked PBS and plasma at all bacterial concentrations (Table S5). This suggests that there was no significant effect of potential inhibitors in plasma that were used for testing other assays.

### Initial clinical evaluation of real-time PCRs

**FIG 3:**
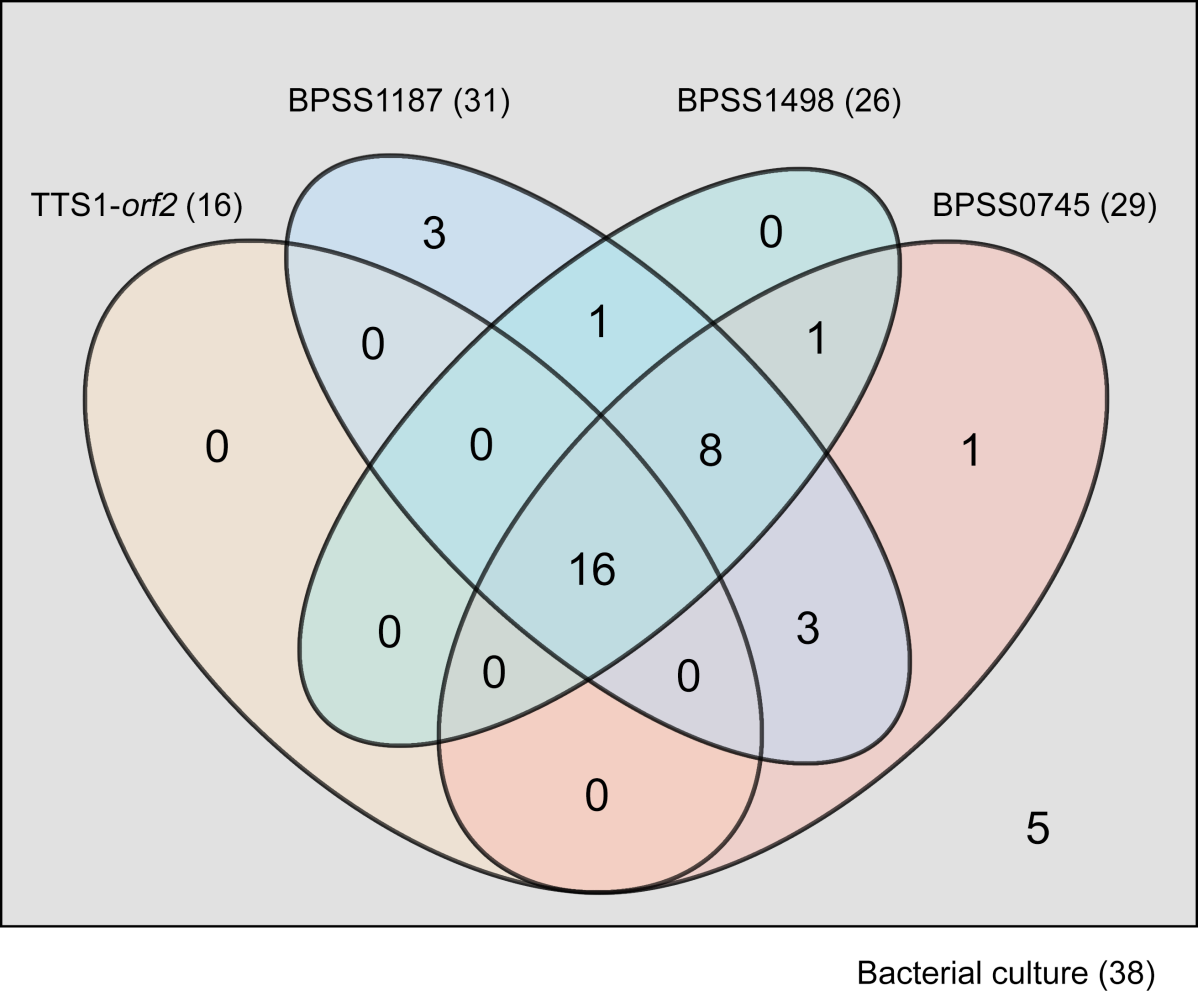
Venn diagram represents the sensitivity of four PCR targets and bacterial cultures for the detection of *B. pseudomallei* in blood samples from 38 patients with bacteremic melioidosis. Four target genes (TTS1-*orf2*, BPSS1187, BPSS1498, and BPSS0745) were initially evaluated using blood samples from 38 patients with bacteremic melioidosis.

Initial clinical evaluation of real-time PCR of TTS1-*orf2*, BPSS0745, BPSS1187, and BPSS1498 was performed with a total of 38 plasma samples collected from 38 patients with bacteremia melioidosis on the day of admission (before culture results were obtained) (Fig. S1C). Melioidosis was subsequently confirmed by bacterial culture. The median time to obtain culture results was 58 h (interquartile range 43–66 h). The results of all real-time PCRs for individual samples are shown in Table S6 and summarized in Table 2. The sensitivity of the BPSS1187 assay was the highest at 81.6%, followed by BPSS0745, BPSS1498, and TTS1-*orf2* at 76.3%, 68.4%, and 42.1%, respectively (Table 2). Of these 38 plasma samples, 16 samples (42%) were positive in all assays, whereas 3 samples (7.9%) were positive by BPSS1187 real-time PCR alone, and 1 sample was positive by BPSS0745 real-time PCR alone. None of these samples were positive for TTS1-*orf2* or BPSS1498 alone (Fig. 3).

**TABLE 2:** Sensitivity and specificity of real-time PCR assays for detecting four DNA targets of *B. pseudomallei* in plasma samples of bacteremic melioidosis patients (*N* = 38) and non-melioidosis patients^*a*^ (*N* = 49)

Assay performance	Real-time PCR assays			
	TTS1-*orf2*	BPSS0745	BPSS1187	BPSS1498
Sensitivity	42.1% (16/38)	76.3% (29/38)	81.6% (31/38)	68.4% (26/38)
Specificity	100% (0/49)	100% (0/49)	100% (0/49)	100% (0/49)

^*a*^ The assays were performed using 4 µL of DNA. Values for sensitivity and specificity are given as the percentage of patients positive (sensitivity) or negative (specificity) by real-time PCRs compared to those of blood cultures. Values in parenthesis indicate positive values out of the total number of patients.

The specificity of real-time PCR assays was evaluated in the plasma of 49 non-melioidosis patients, of whom 28 (57.1%) presented bacteremia. The patients had laboratory-confirmed other pathogenic infections, including *Acinetobacter baumanii* (*N* = 3), *Aeromonas hydrophila* (*N* = 1), *Brucella* spp. (*N* = 1), *Corynebacterium* spp. (*N* = 1), *Enterobacter cloacae* (*N* = 2), *Enterococcus faecalis* (*N* = 5), *Enterococcus faecium* (*N* = 1), *Escherichia coli* (*N* = 17), *Klebsiella pneumoniae* (*N* = 4), *Pseudomonas aeruginosa* (*N* = 1), *Staphylococcus aureus* (*N* = 2), *Streptococcus pyogenes* (*N* = 2), and mixed infections (*N* = 9). None of the DNA extracts from these plasma samples were positive in any of the four assays (100% specificity for all assays).

### Prospective clinical evaluation of BPSS1187 and TTS1-*orf2* real-time PCRs (the second phase)

BPSS1187 and TTS1-*orf2* were successfully used for *B. pseudomallei* detection in soil samples in areas where melioidosis is endemic (21, 29). Since BPSS1187 real-time PCR showed the highest sensitivity in our study, we prospectively compared the assay performance between BPSS1187 and the most widely used TTS1*-orf2* real-time PCR (21, 37, 38) for detection of *B. pseudomallei* DNA from clinical samples, using both patients and specimen types as denominators. The evaluation was conducted at two hospitals (Mukdahan and Roi Et Hospitals) in Northeast Thailand (Table 3). Five milliliters of blood samples were obtained from 421 suspected patients with melioidosis, of whom 77 (18.2%) were positive for *B. pseudomallei* by bacterial culture in one or more clinical samples, and 99 (23.5%) were patients with other infections (Table S7). Two hundred and forty-five patients were excluded from further analysis as any pathogenic organisms were not found in the bacterial culture.

**TABLE 3:** Sensitivity and specificity of TTS1-*orf2* and BPSS1187 real-time PCRs for *B. pseudomallei* detection in comparison with bacterial culture using the number of patients as the denominator^*a*^

Patients	%Sensitivity (95% CI)			%Specificity (95% CI)		
	TTS1-*orf2*	BPSS1187	Combined TTS1-*orf2* and BPSS1187	TTS1-*orf2*	BPSS1187	Combined TTS1-*orf2* and BPSS1187
**Mukdahan Hospital**	26/32 81.3% (64.7–91.1)	32/32 100% (89.3–100)	32/32 100% (89.3–100)	55/56 98.2% (90.6–99.9)	53/56 94.6% (85.4–98.5)	52/56 92.9% (83.0–97.2)
Melioidosis (*N* = 32)						
Other infections (*N* = 56)						
Total (*N* = 88)						
**Roi Et Hospital**	35/45 77.8% (63.7–87.5)	37/45 82.2% (68.7–90.7)	39/45 86.7% (73.8–93.7)	43/43 100% (91.8–100)	42/43 97.7% (87.9–99.9)	42/43 97.7% (87.9–99.9)
Melioidosis (*N* = 45)						
Other infections (*N* = 43)						
Total (*N* = 88)						
**Overall**	61/77 79.2% (68.9–86.8)	69/77 89.6% (80.8–94.6)	71/77 92.2% (84.0–96.4)	98/99 99% (94.5–99.9)	95/99 96% (90.1–98.4)	94/99 94.9% (88.7–97.8)
Melioidosis (*N* = 77)						
Other infections (*N* = 99)						
Total (*N* = 176)						

^*a*^ Real-time PCRs were performed on all clinical samples. The assays were prospectively evaluated in 176 suspected patients with melioidosis from Mukdahan and Roi Et Hospitals, including 77 culture-confirmed melioidosis patients and 99 patients with other pathogenic infections. Values for sensitivity and specificity are shown as the number of positive and negative samples, respectively, by real-time PCR out of the total number of samples tested. Values in parenthesis indicate the percentage of positives for sensitivity and the percentage of negatives for specificity. CI, confidence interval.

Using the patient as the denominator, BPSS1187 real-time PCR showed higher sensitivity than TTS1-*orf2* real-time PCR assay {89.6% [95% confidence interval (CI), 80.8%–94.6%] versus 79.2% [95% CI, 68.9%–86.8%], *P* = 0.004}. The specificity was comparable between BPSS1187 and TTS1-*orf2* [96.0 (95% CI, 90.1–98.4) versus 99.0 (95% CI, 94.5–99.9), *P* = 0.375]. All samples were detected in the acceptable range of Ct values for TTS1-Ava internal control (Fig. S1E). Similar results were observed between different PCR thermocyclers (Table S8) and the clinical evaluations from the two hospitals (Table 3; Table S9).

**FIG 4:**
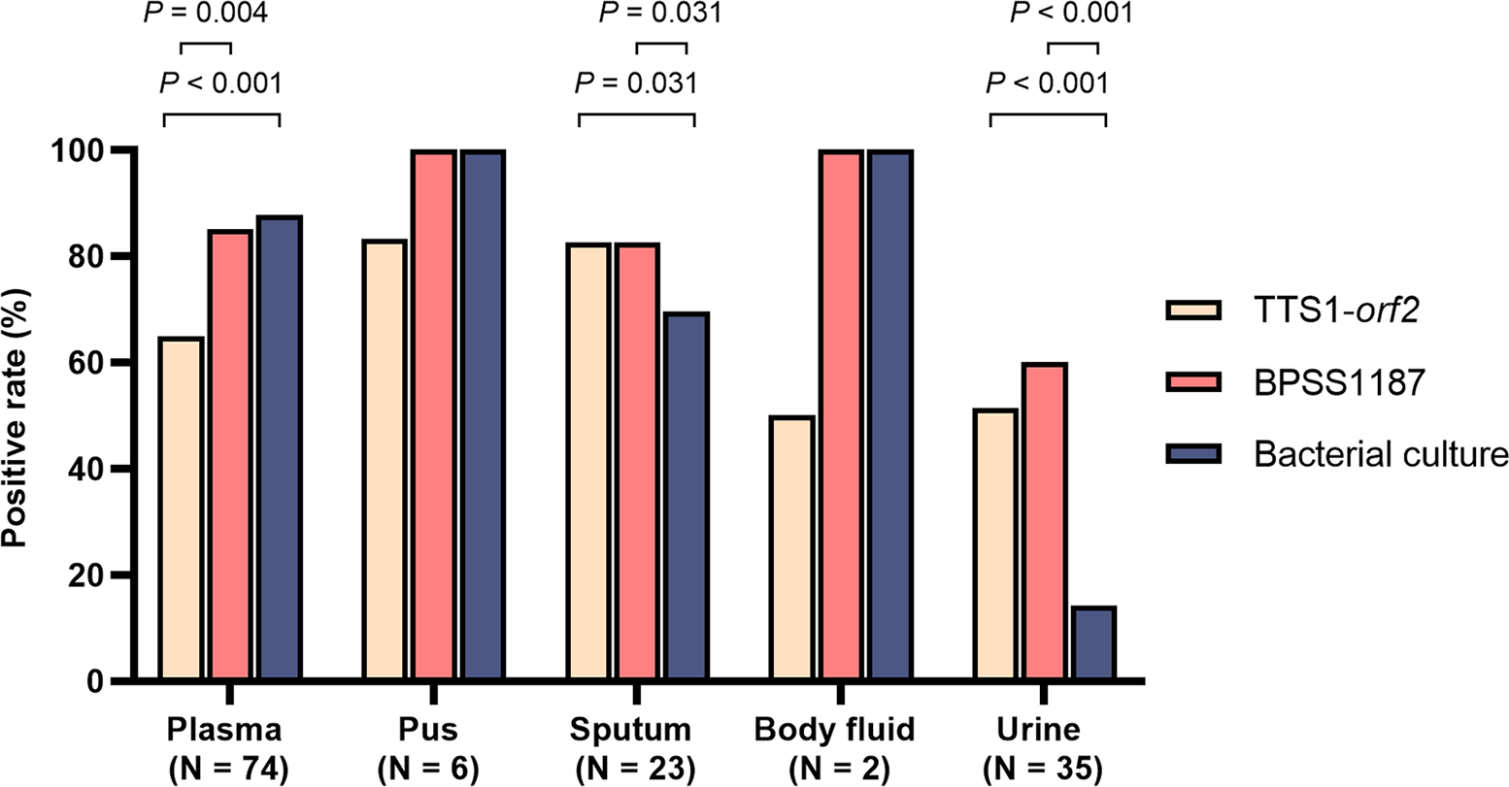
Positive rate of TTS1-*orf2* and BPSS1187 real-time PCRs and culture in different clinical samples from patients with culture-confirmed melioidosis (*N* = 140).

The second evaluation was performed using specimen type as the denominator. Multiple clinical samples (*N* = 140), including plasma (*N* = 74), pus (*N* = 6), sputum (*N* = 23), body fluid (*N* = 2), and urine (*N* = 35) from 77 patients with melioidosis, were used for conducting TTS1-*orf2* and BPSS1187 real-time PCRs and bacterial cultures. The positive rate of real-time PCR was greater than that of bacterial culture in the detection of *B. pseudomallei* in sputum [both TTS1-*orf2* and BPSS1187 (82.6%) versus bacterial culture (69.6%), *P* = 0.031] and urine [TTS1-*orf2* (51.4%) versus culture (14.3%), *P* < 0.001; BPSS1187 (60%) versus culture (14.3%), *P* < 0.001]. BPSS1187 assay demonstrated a higher detection rate than TTS1-*orf2* in plasma [BPSS1187 (85.1%) versus TTS1-*orf2* (64.9%), *P* = 0.004] (Fig. 4; Table S9).

The comparison of sensitivity and specificity was also performed using the specimen type as the denominator (Table 4). Of the 91 clinical specimens that were culture positive for *B. pseudomallei*, TTS1-*orf2* and BPSS1187 real-time PCRs were positive for 70.1% and 83.5% of the specimens, respectively (*P* = 0.002). Of the 85 specimens that were culture positive for other bacterial pathogens, TTS1-*orf2* real-time PCR and BPSS1187 real-time PCR were negative for 100% and 98.8% of the specimens, respectively (*P* = 1.000). The only sample with other bacterial infection that was false positive by BPSS1187 real-time PCR was sputum with *Klebsiella pneumoniae*.

**TABLE 4:** Sensitivity and specificity of TTS1-*orf2* and BPSS1187 real-time PCRs compared with those of culture for *B. pseudomallei* in melioidosis (*N* = 91) and patients with other bacterial infections (*N* = 85) using sample type as the denominator^*a*^

Sample type	Sensitivity			Specificity		
	TTS1-*orf2*	BPSS1187	*P* value	TTS1-*orf2*	BPSS1187	*P* value
Plasma	44/65 (67.7%)	55/65 (84.6%)	0.007	41/41 (100%)	41/41 (100%)	1.000
Pus	5/6 (83.3%)	6/6 (100%)	1.000	7/7 (100%)	7/7 (100%)	1.000
Sputum	13/13 (100%)	13/13 (100%)	1.000	21/21 (100%)	20/21 (95.2%)	1.000
Body fluid	1/2 (50%)	2/2 (100%)	1.000	1/1 (100%)	1/1 (100%)	1.000
Urine	5/5 (100%)	5/5 (100%)	1.000	15/15 (100%)	15/15 (100%)	1.000
Total	68/91 (70.1%)	81/91 (83.5%)	0.002	85/85 (100%)	84/85 (98.8%)	1.000

^*a*^ Values for sensitivity and specificity are shown as the number of positive and negative samples, respectively, by real-time PCR out of the total number of samples tested. Values in parenthesis indicate the percentage of positives for sensitivity and the percentage of negatives for specificity.

## DISCUSSION

Early diagnosis is critical for the implementation of effective antibiotic treatments to inhibit the progression of disease and prevent death. The identification of *B. pseudomallei* in clinical samples in endemic areas relies on culture-based methods, but they have poor sensitivity. Molecular-based diagnostic methods need to be sensitive and specific for all sample types. However, the current PCR methods are not sensitive to blood samples (18). This study developed an optimal method for the DNA extraction of *B. pseudomallei* and used internal control for real-time PCRs. We evaluated the real-time PCRs developed in previous studies [TTS1-*orf2* (25), BPSS0745 (21), BPSS0087 (21), BPSS1187 (22), and BPSS1492 (*bimA*) (26)] and this study [BPSS1498 (*hcp1*)]. We identified BPSS1187 as the best PCR target gene for early detection of *B. pseudomallei* infection in patients before the culture results were obtained.

The concentration of bacteria in different sample types is critical for the detection of *B. pseudomallei*. Previous studies have reported that *B. pseudomallei* detection by PCR in blood samples (200 µL) had less sensitivity than that in other clinical samples, such as sputum, urine, or pus. This is related to the variable quantities of bacteria in different clinical specimens. The median concentration of *B. pseudomallei* in blood was previously reported to be 1.1 CFU/mL; in contrast, other specimens were reported to contain a high bacterial load from localized sites of infection, such as 1.5 × 10^4^ CFU/mL in urine, 1.1 × 10^5^ CFU/mL in sputum, and 1.1 × 10^7^ CFU/mL in pus (11, 37, 38). Importantly, the mortality rate was positively correlated with bacterial quantities in blood and urine samples. The mortality rate of patients with *B. pseudomallei* count in blood >100 CFU/mL was 96%, while the rate for bacterial count in blood ≤1 CFU/mL was 42% (39). The mortality rate of patients with *B. pseudomallei* count in urine pellets ≥10^5^ CFU/mL was 71%, while the rate for bacterial count in urine pellets <10^3^ CFU/mL was 58% (40). This study demonstrated that DNA extraction from high-volume plasma samples could increase the sensitivity of real-time PCR. The observed increase in sensitivity might be influenced by a higher bacterial concentration. Although *B. pseudomallei* was intracellular bacteria, we demonstrated that plasma could be used for DNA extraction for real-time PCR. Whole blood or buffy coat may be used for DNA extraction, but extraction from plasma fraction showed an advantage over whole blood or buffy coat when using the QIAamp DNA Mini Kit with a low elution volume of DNA (41). The high sensitivity with a high volume of plasma sample might be due to the increased concentration of DNA and the low amount of PCR inhibitor (42).

The selection of gene targets is important for real-time PCR development. *In silico* analysis showed that all six genes were present in *B. pseudomallei* isolates. All primers and probes could detect all of the *B. pseudomallei* tested, although some variations were observed for these regions. This enables the broad application of these real-time PCR assays to diverse genotypes. The TTS1-*orf2* real-time PCR was previously reported to elicit false negatives in the detection of soil samples from Northeast Thailand, and the sensitivity improved when BPSS1187 nested PCR detection was used (29). Nested PCR involves an additional round of amplification. It may not be practical for routine use in clinical settings in low- and middle-income countries due to the cost implications and the technical requirements to minimize the risk of contamination. The TTS1-*orf2* assay was also used in combination with BPSS0745 to enhance the detection rate of *B. pseudomallei* in soils from 76.5% to 90% (21). However, our clinical evaluation showed that BPSS1187 real-time PCR was the most sensitive method compared with the real-time PCRs of other targets, including TTS1-*orf2*, BPSS0745, BPSS1498, BPSS0087, and BPSS1492. Thus, the multiple gene targets using genes evaluated in this study may not increase the sensitivity of the real-time PCR assay significantly. It should be noted that our study was designed to screen and select a simple single target using SYBR Green I real-time PCR. However, other PCR targets, such as BPSS0087 and BPSS1492, were not included in the subsequent clinical evaluation process using TagMan real-time PCR. These targets may demonstrate similar performance to those analyzed. Future studies should be conducted to explore and evaluate new PCR targets that might enhance the detection of *B. pseudomallei* in clinical samples, particularly when combined with the BPSS1187 target.

The difference in real-time PCR sensitivity between initial evaluation (Table 2) and second-phase evaluation (Tables 3 and 4) was noted. This might be due to some differences between the two melioidosis cohorts. In the first cohort, we evaluated real-time PCRs in the plasma of bacteremic melioidosis patients at one hospital, while in the second cohort, we evaluated melioidosis patients who were *B. pseudomallei* culture positive from any specimens in two hospitals. The lower sensitivity of real-time PCRs for plasma samples (Table 4) was likely associated with a lower bacterial concentration. However, we did not perform viable bacterial counts on all specimens used in this study.

In previous studies in Thailand, real-time PCRs for the diagnosis of melioidosis in blood samples were reported to be insensitive. However, this study has made significant improvements in *B. pseudomallei* DNA detection, particularly in plasma samples where bacteremia upon admission occurs in the majority of all patients (3). In comparison with the findings of Noparatvarakorn et al. (20) in Thailand, which showed a sensitivity of 34.6% in plasma and 78.2% in all clinical samples (20), and Chantratita et al. (38), which reported a sensitivity of 0% in buffy coat samples and an overall sensitivity of 25.9% across all samples (38), our optimized real-time PCR method targeting the BPSS1187 gene exhibited higher sensitivities, with 84.6% sensitivity in plasma and 83.5% in all clinical specimens.

BPSS1187 real-time PCR assay for *B. pseudomallei* detection in clinical samples provided a shorter turnaround time. The assay showed higher sensitivity than bacterial culture in sputum and urine samples. This diagnostic tool is critical for patient survival, especially in patients with septicemic melioidosis, of whom 45% of blood samples contain less than 1 CFU/mL (39). A previous report demonstrated that the BPSS1187 target was 100% positive in blood samples from all seven fatal melioidosis cases evaluated in hospitalized patients with septicemia in a hospital in Northeast Thailand (22). Compared to our study, the sensitivity of BPSS1187 in the previous study was 71%. Our study showed that BPSS1187 real-time PCR could not detect *B. pseudomallei* in approximately 12% of patients with melioidosis. Among these, one patient died from melioidosis. The DNA may not be detectable in some clinical specimens or during later phases of infection. Indeed, we observed variable results for real-time PCRs in different specimen types. Therefore, if real-time PCR results were negative for patients with suspected melioidosis, culture results should be used for further confirmation. Serological assays such as enzyme-linked immunosorbent assays may be useful (24, 43). However, the lack of antibodies in acute illness and the persistence of antibodies in patients with a previous history of melioidosis should be considered for interpretation.

Molecular testing showed superior performance for *B. pseudomallei* detection compared to bacterial culture in sputum and urine samples. The bacterial culture results from the two study hospitals may vary due to differences in laboratory personnel and the routine use of different equipment platforms. Bacterial culture could lead to false-negative results in patients with low quantities of bacteria in clinical samples and may be associated with the commencement of antibiotic treatment prior to sample collection. Negative urine culture results could occur in 25%–30% of symptomatic women with an uncomplicated urinary tract infection (44, 45). False negative results of urine cultures were demonstrated; for example, PCR detection of *Escherichia coli* was 90% positive in the urine samples of 42 culture-negative symptomatic women (46). Centrifugation or filtration is required to enhance the detection rate when urine samples contain low amounts of bacterial pathogens (47). Our study used a bacterial pellet from a 10-mL centrifuged urine sample for DNA extraction to achieve a 5/5 (100%) PCR positive for *B. pseudomallei* culture-positive urine samples. However, standard routine culture in the hospital laboratory used a calibrated inoculating loop that picked up 1 µL of uncentrifuged urine samples. False negatives by culture might result from very low amounts of *B. pseudomallei* in patients with mild infections or mixed infections. No detection or misidentification of *B. pseudomallei* in sputum culture by PCR or culture might be caused by the outgrowth of other bacteria, such as normal respiratory flora or other pathogens colonized in the respiratory tract (48) because *B. pseudomallei* grows more slowly than many other organisms (2).

In this study, we used 2.5 pg TTS1-Ava DNA as an internal control to ensure the accuracy, reliability, and effectiveness of the PCR operational processes. However, an amount of DNA was lost during extraction and real-time PCR. Despite this, we still detected the TTS1-Ava DNA control in the PCR reactions. There are several advantages to internal control for designing and implementing a PCR experiment, mainly in checking for differences in DNA extraction efficiency and the presence of PCR inhibitors (49). Internal control measures help reduce the risk of errors and improve the accuracy of PCR results. Implementing internal control measures can help streamline PCR experiments and reduce the time and resources required to obtain reliable results. For example, the addition of *Bacillus thuringiensis* to multiplex qPCR for *Burkholderia mallei* and *B. pseudomallei* helps to decrease false-negative results (50). This can help increase confidence in the data and support more robust decision-making.

A limitation of molecular tests is that positive PCR detection could not help distinguish between living and dead bacteria (51, 52), limiting their use for monitoring infection. However, real-time PCR may be used in combination with bacterial culture to monitor the success of treatment. It is possible that dead bacteria are eliminated more rapidly than live and replicating bacteria, but this hypothesis needs more studies for confirmation in melioidosis. Based on the findings in this study, BPSS1187 was more sensitive than other real-time PCR targets. Single BPSS1187 real-time PCR detecting DNA extracted from high volumes of plasma, urine, and other clinical samples demonstrated high sensitivity and specificity by real-time PCR assay. The median time to obtain culture results was 58 h, while the time for PCR was 3–4 h. To avoid delays in treatment of melioidosis that may lead to death, we suggest using this approach for routine early detection of *B. pseudomallei* in clinical settings in endemic regions of melioidosis.

## SUPPLEMENTAL MATERIAL

Figure S1 spectrum.01039-23-s0001.docx Figure S1: Overview of the study.

Table S1 spectrum.01039-23-s0002.xlsx Table S1: Information of *B. pseudomallei* clinical isolates (*N* = 1,294).

Table S2 spectrum.01039-23-s0003.docx Table S2: List of *Burkholderia pseudomallei* strains used for evaluation of target sensitivity (*N* = 31).

Table S3 spectrum.01039-23-s0004.docx Table S3: Variation and proportion of each real-time PCR target sequences.

Table S4 spectrum.01039-23-s0005.docx Table S4: Limit of detection (LOD) of each real-time PCR target.

Table S5 spectrum.01039-23-s0006.docx Table S5: Comparison of *B. pseudomallei* detection by TTS1-orf2 real-time PCR in *B. pseudomallei*-spiked PBS and *B. pseudomallei*-spiked plasma.

Table S6 spectrum.01039-23-s0007.docx Table S6: Real-time PCR results of individual samples from patients (*N* = 87).

Table S7 spectrum.01039-23-s0008.docx Table S7: Organisms isolated from clinical samples of 99 other infection patients.

Table S8 spectrum.01039-23-s0009.docx Table S8: Comparison of the BPSS1187 real-time PCR assay (*N* = 19) between the CFX96 Touch Real-Time PCR system (Bio-Rad) and the Quantstudio 1 Real-Time PCR system (Applied Biosystems) with FAM and HEX fluorescence signals.

Table S9 spectrum.01039-23-s0010.docx Table S9: Real-time PCRs and culture results of clinical samples from melioidosis patients (*N* = 140).

ASM does not own the copyrights to Supplemental Material that may be linked to, or accessed through, an article. The authors have granted ASM a non-exclusive, world-wide license to publish the Supplemental Material files. Please contact the corresponding author directly for reuse.
