# Supplementary material for: Optimization and prospective evaluation of sensitive real-time PCR assays with an internal control for the diagnosis of melioidosis in Thailand
Source: Microbiol Spectr. 2023 Oct 11;11(6):e01039-23. doi: 10.1128/spectrum.01039-23 (PMC10715024; doi:10.1128/spectrum.01039-23)
Supplement: Figure S1 — Overview of the study. [file spectrum.01039-23-s0001.docx]

**
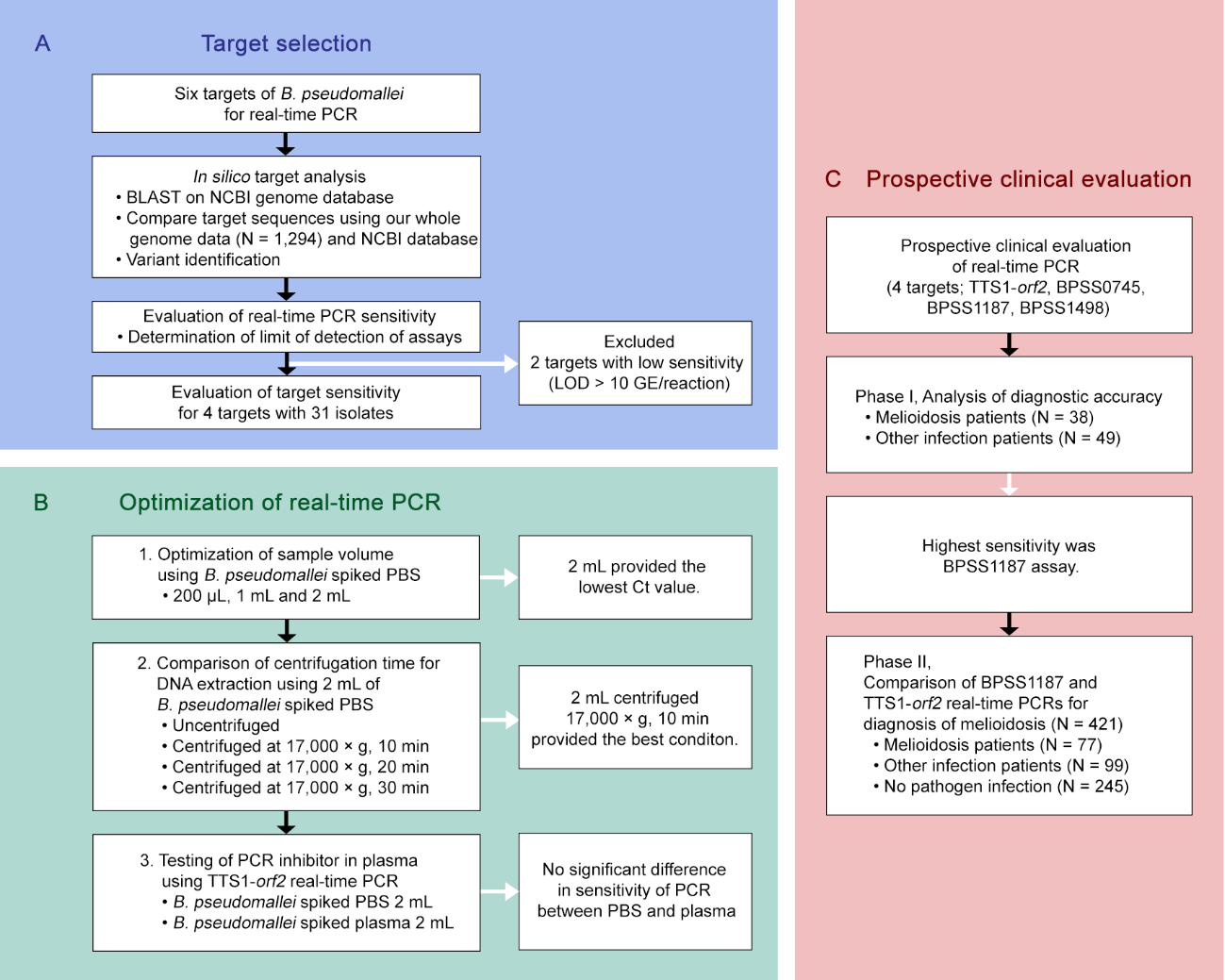
**

**

**

**
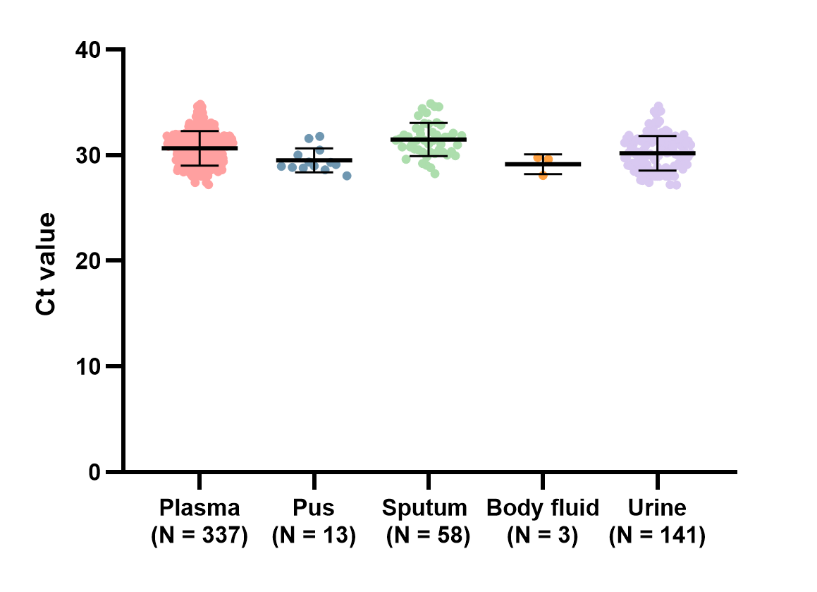
E**

| **Type of clinical samples** | **Number of samples** | **Mean Ct value** | **SD** |
| --- | --- | --- | --- |
| Plasma | 337 | 30.65 | 1.62 |
| Pus | 13 | 29.53 | 1.13 |
| Sputum | 58 | 31.47 | 1.57 |
| Body fluid | 3 | 29.16 | 0.94 |
| Urine | 141 | 30.19 | 1.64 |

**Figure S1:** Overview of the study. Real-time PCR assays have been developed for the rapid detection of *B. pseudomallei* DNA. The assays were analyzed for target selection (A) and optimization experiments were performed. One-way analysis of variance was used to determine the reproducibility of DNA extraction conditions. Tukey multiple comparisons test was used to compare the Ct values of the paired groups. (B). Four target genes were selected for clinical evaluation for melioidosis diagnosis. The sensitivity and specificity were calculated using bacterial culture results as a gold standard. The McNemar test was used to compare the sensitivity and specificity between tests. (C). Range of linearity for four *B. pseudomallei* real-time PCR assays (D). Ct values of internal control presented in various samples of patients (N = 140) (E).
