# Supplementary material for: Optimization and prospective evaluation of sensitive real-time PCR assays with an internal control for the diagnosis of melioidosis in Thailand
Source: Microbiol Spectr. 2023 Oct 11;11(6):e01039-23. doi: 10.1128/spectrum.01039-23 (PMC10715024; doi:10.1128/spectrum.01039-23)
Supplement: Table S2 — List of Burkholderia pseudomallei strains used for evaluation of target sensitivity (N = 31). [file spectrum.01039-23-s0003.docx]

**Table S2:** List of *Burkholderia pseudomallei* strains used for evaluation of target sensitivity

(N = 31)

| **Bacteria** | **Strain** | **Origin** | **Source** | **Target** | **Group** | **Ct Value** |
| --- | --- | --- | --- | --- | --- | --- |
| *B. pseudomallei* | DR10012A | Thailand | Clinical | TTS1-*orf2* | 1 | 15.53 |
| *B. pseudomallei* | DR10095A | Thailand | Clinical | TTS1-*orf2* | 1 | 14.72 |
| *B. pseudomallei* | DR10109A | Thailand | Clinical | TTS1-*orf2* | 1 | 14.62 |
| *B. pseudomallei* | DR20027A | Thailand | Clinical | TTS1-*orf2* | 2 | 13.85 |
| *B. pseudomallei* | DR80175A | Thailand | Clinical | BPSS0745 | 1 | 16.23 |
| *B. pseudomallei* | DR90084A | Thailand | Clinical | BPSS0745 | 1 | 14.89 |
| *B. pseudomallei* | DR90080A | Thailand | Clinical | BPSS0745 | 1 | 15.35 |
| *B. pseudomallei* | DR20085A | Thailand | Clinical | BPSS0745 | 2 | 16.31 |
| *B. pseudomallei* | DR20003A | Thailand | Clinical | BPSS0745 | 3 | 15.66 |
| *B. pseudomallei* | DR20029A | Thailand | Clinical | BPSS0745 | 3 | 15.11 |
| *B. pseudomallei* | DR40111A | Thailand | Clinical | BPSS0745 | 3 | 16.65 |
| *B. pseudomallei* | DR90086A | Thailand | Clinical | BPSS0745 | 4 | 15.15 |
| *B. pseudomallei* | DR20023A | Thailand | Clinical | BPSS1187 | 1 | 17.17 |
| *B. pseudomallei* | DR20047A | Thailand | Clinical | BPSS1187 | 1 | 16.93 |
| *B. pseudomallei* | DR30020A | Thailand | Clinical | BPSS1187 | 1 | 17.63 |
| *B. pseudomallei* | DR50114A | Thailand | Clinical | BPSS1187 | 2 | 16.97 |
| *B. pseudomallei* | DR60066A | Thailand | Clinical | BPSS1187 | 2 | 16.92 |
| *B. pseudomallei* | DR70040A | Thailand | Clinical | BPSS1187 | 2 | 16.60 |
| *B. pseudomallei* | DR10174A | Thailand | Clinical | BPSS1498 | 1 | 16.78 |
| *B. pseudomallei* | DR50193A | Thailand | Clinical | BPSS1498 | 1 | 16.18 |
| *B. pseudomallei* | DR80134A | Thailand | Clinical | BPSS1498 | 1 | 15.79 |
| *B. pseudomallei* | DR40128A | Thailand | Clinical | BPSS1498 | 2 | 16.44 |
| *B. pseudomallei* | DR50173A | Thailand | Clinical | BPSS1498 | 2 | 15.73 |
| *B. pseudomallei* | DR70003A | Thailand | Clinical | BPSS1498 | 2 | 16.39 |
| *B. pseudomallei* | DR10094A | Thailand | Clinical | BPSS1498 | 3 | 16.74 |
| *B. pseudomallei* | DR10111A | Thailand | Clinical | BPSS1498 | 4 | 17.52 |
| *B. pseudomallei* | DR40032A | Thailand | Clinical | BPSS1498 | 4 | 16.48 |
| *B. pseudomallei* | DR90103A | Thailand | Clinical | BPSS1498 | 4 | 16.38 |
| *B. pseudomallei* | DR50065A | Thailand | Clinical | BPSS1498 | 5 | 17.21 |
| *B. pseudomallei* | DR60060A | Thailand | Clinical | BPSS1498 | 5 | 17.54 |
| *B. pseudomallei* | DR80051A | Thailand | Clinical | BPSS1498 | 5 | 17.19 |
