# Supplementary material for: Optimization and prospective evaluation of sensitive real-time PCR assays with an internal control for the diagnosis of melioidosis in Thailand
Source: Microbiol Spectr. 2023 Oct 11;11(6):e01039-23. doi: 10.1128/spectrum.01039-23 (PMC10715024; doi:10.1128/spectrum.01039-23)
Supplement: Table S3 — Variation and proportion of each real-time PCR target sequences. [file spectrum.01039-23-s0004.docx]

**Table S3:** Variation and proportion of each real-time PCR target sequences

| **PCR target** | **Group** | **SNP location (chr 2) of Genbank accession number BX571966** | **Genome data**  **(Total 1,294 isolates)** | **NCBI**  **(Number of isolates)*^a^*** | **Total** |
| --- | --- | --- | --- | --- | --- |
| TTS1-*orf2*  (115 bp) | 1 | - | 1293 (99.9%) | 149/149 (100%) | 1442/1443 (99.9%) |
|  | 2 | 1920755 | 1 (0.1%) | - | 1/1443 (0.1%) |
| BPSS0745  (132 bp) | 1 | - | 679 (52.5%) | 109/141 (77.3%) | 788/1435 (54.9%) |
|  | 2 | 1002098, 1002160 | 1 (0.1%) | - | 1/1435 (0.1%) |
|  | 3 | 1002160 | 613 (47.4%) | 32/141 (22.7%) | 645/1435 (44.9%) |
|  | 4 | 1002040 | 1 (0.1%) | - | 1/1435 (0.1%) |
| BPSS1187  (81 bp) | 1 | - | 956 (73.9%) | 106/143 (74.1%) | 1062/1437 (73.9%) |
|  | 2 | 1593855 | 338 (26.1%) | 33/143 (23.1%) | 371/1437 (25.8%) |
| BPSS1187  (81 bp) | 3 | 1593912 | - | 1/143 (0.7%) | 1/1437 (0.1%) |
|  | 4 | 1593855, 1593912 | - | 1/143 (0.7%) | 1/1437 (0.1%) |
|  | 5 | 1593880 | - | 1/143 (0.7%) | 1/1437 (0.1%) |
|  | 6 | 1593870 | - | 1/143 (0.7%) | 1/1437 (0.1%) |
| BPSS1498  (*hcp1*)  (157 bp) | 1 | - | 1219 (94.2%) | 81/175 (46.3%) | 1300/1469 (88.5%) |
|  | 2 | 2043177 | 40 (3.1%) | 76/175 (43.4%) | 116/1469 (7.9%) |
|  | 3 | 2043216 | 1 (0.1%) | 3/175 (1.7%) | 4/1469 (0.3%) |
| BPSS1498  (*hcp1*)  (157 bp) | 4 | 2043148, 2043177, 2043182, 2043186, 2043192, 2043199, 2043207, 2043232, 2043302 | 10 (0.8%) | 5/175 (2.9%) | 15/1469 (1%) |
|  | 5 | 2043148, 2043177, 2043182, 2043186, 2043192, 2043199, 2043207, 2043231, 2043232, 2043302 | 14 (1.1%) | - | 14/1469 (1%) |
|  | 6 | 2043218 | - | 2/175 (1.1%) | 2/1469 (0.1%) |
|  | 7 | 2043177 | - | 2/175 (1.1%) | 2/1469 (0.1%) |
|  | 8 | 2043210 | - | 6/175 (3.4%) | 6/1469 (0.4%) |
| BPSS0087  (88 bp) | 1 | - | 1000 (77.3%) | 100/144 (88.6%) | 1100/1438 (76.5%) |
| BPSS0087  (88 bp) | 2 | 102558 | 294 (22.7%) | 37/144 (25.7%) | 331/1438 (23%) |
|  | 3 | 102560 | - | 3/144 (2.1%) | 3/1438 (0.2%) |
|  | 4 | 102564 | - | 1/144 (0.7%) | 1/1438 (0.1%) |
|  | 5 | 102589 | - | 2/144 (1.4%) | 2/1438 (0.1%) |
|  | 6 | 102546 | - | 1/144 (0.7%) | 1/1438 (0.1%) |
| BPSS1492  (*bimA*)  (122 bp) | 1 | - | 994 (76.8%) | 116/131 (88.6%) | 1110/1425 (77.9%) |
|  | 2 | 2034251, 2034293, 2034306 | 291 (22.5%) | 10/131 (7.6%) | 301/1425 (21.1%) |
|  | 3 | 2034306 | 8 (0.6%) | - | 8/1425 (0.6%) |
| BPSS1492  (*bimA*)  (122 bp) | 4 | 2034221 | 1 (0.1%) | - | 1/1425 (0.1%) |
|  | 5 | 2034291 | - | 2/131 (1.5%) | 2/1425 (0.1%) |
|  | 6 | 2034240 | - | 1/131 (0.8%) | 1/1425 (0.1%) |
|  | 7 | 2034210, 2034251, 2034306 | - | 2/131 (1.6%) | 2/1425 (0.1%) |

*^a^*Total number of bacterial isolates was presented as all sequences that produced significant alignments from BLASTn searches of NCBI database.
