# Supplementary material for: Optimization and prospective evaluation of sensitive real-time PCR assays with an internal control for the diagnosis of melioidosis in Thailand
Source: Microbiol Spectr. 2023 Oct 11;11(6):e01039-23. doi: 10.1128/spectrum.01039-23 (PMC10715024; doi:10.1128/spectrum.01039-23)
Supplement: Table S4 — Limit of detection (LOD) of each real-time PCR target. [file spectrum.01039-23-s0005.docx]

**Table S4:** Limit of detection (LOD) of each real-time PCR target

| **DNA** | **Amount of DNA (GE/reaction)** | **TTS1-*orf2*** | | | **BPSS0745** | | | **BPSS1187** | | | **BPSS1498** | | |
| --- | --- | --- | --- | --- | --- | --- | --- | --- | --- | --- | --- | --- | --- |
|  |  | **No. of amplified** | **Mean C_T_** | **SD** | **No. of amplified** | **Mean C_T_** | **SD** | **No. of amplified** | **Mean C_T_** | **SD** | **No. of amplified** | **Mean C_T_** | **SD** |
| *B. pseudomallei* | 1x10^6^ | 9/9 | 15.69 | 0.15 | 9/9 | 15.96 | 0.24 | 9/9 | 19.02 | 0.23 | 9/9 | 16.26 | 0.28 |
| *B. pseudomallei* | 1x10^5^ | 9/9 | 18.91 | 0.23 | 9/9 | 19.01 | 0.24 | 9/9 | 22.21 | 0.23 | 9/9 | 19.53 | 0.28 |
| *B. pseudomallei* | 1x10^4^ | 9/9 | 22.43 | 0.24 | 9/9 | 22.59 | 0.29 | 9/9 | 25.59 | 0.21 | 9/9 | 23.29 | 0.23 |
| *B. pseudomallei* | 1x10^3^ | 9/9 | 27.29 | 0.80 | 9/9 | 27.51 | 0.72 | 9/9 | 30.08 | 0.55 | 9/9 | 28.32 | 0.45 |
| *B. pseudomallei* | 1x10^2^ | 12/12 | 31.20 | 0.28 | 12/12 | 31.26 | 0.42 | 12/12 | 33.85 | 0.65 | 12/12 | 32.27 | 0.61 |
| *B. pseudomallei* | 1x10^1^ | 11/12 | 34.36 | 1.1 | 12/12 | 35.29 | 1.4 | 11/12 | 38.39 | 1.4 | 11/12 | 37.26 | 1.8 |
| *B. pseudomallei* | 1 x10^0^ | 3/12 | 37.02 | 0.52 | 4/12 | 36.09 | 0.31 | 1/12 | 41.77 | 0 | 0/12 | - | - |

| **DNA** | **Amount of DNA (GE/reaction)** | **BPSS0087** | | | **BPSS1492** | | |
| --- | --- | --- | --- | --- | --- | --- | --- |
|  |  | **No. of amplified** | **Mean C_T_** | **SD** | **No. of amplified** | **Mean C_T_** | **SD** |
| *B. pseudomallei* | 1x10^6^ | 9/9 | 16.82 | 0.11 | 9/9 | 17.50 | 0.11 |
| *B. pseudomallei* | 1x10^5^ | 9/9 | 20.22 | 0.10 | 9/9 | 21.09 | 0.14 |
| *B. pseudomallei* | 1x10^4^ | 9/9 | 23.89 | 0.12 | 9/9 | 24.96 | 0.19 |
| *B. pseudomallei* | 1x10^3^ | 9/9 | 28.60 | 0.31 | 9/9 | 29.79 | 0.38 |
| *B. pseudomallei* | 1x10^2^ | 12/12 | 34.15 | 0.30 | 12/12 | 35.18 | 0.51 |
| *B. pseudomallei* | 1x10^1^ | 9/12 | 38.40 | 1.4 | 8/12 | 38.30 | 1.2 |
| *B. pseudomallei* | 1 x10^0^ | 2/12 | 39.21 | 0.96 | 0/12 | - |  |

Nine replicates were run for the high DNA concentrations from 1×10^6^ to 1×10^3^ GE/reaction, and 12 replicates were performed for a low concentration (1×10^2^ to 1 GE/reaction). The LOD (yellow highlighted fields) was defined as the concentration at which positive reaction results were observed in 95% or more of the replicated reactions.
