# Supplementary material for: Optimization and prospective evaluation of sensitive real-time PCR assays with an internal control for the diagnosis of melioidosis in Thailand
Source: Microbiol Spectr. 2023 Oct 11;11(6):e01039-23. doi: 10.1128/spectrum.01039-23 (PMC10715024; doi:10.1128/spectrum.01039-23)
Supplement: Table S5 — Comparison of B. pseudomallei detection by TTS1-orf2 real-time PCR in B. pseudomallei-spiked PBS and B. pseudomallei-spiked plasma. [file spectrum.01039-23-s0006.docx]

**Table S5:** Comparison of *B. pseudomallei* detection by TTS1-*orf2* real-time PCR in *B. pseudomallei*-spiked PBS and *B. pseudomallei*-spiked plasma

| ***B. pseudomallei* concentration (CFU/ml)** | **Mean Ct value (SD)** | | ***P* value** |
| --- | --- | --- | --- |
|  | **Spiked PBS** | **Spiked plasma** |  |
| 1×10^6^ | 24.3 (0.37) | 25.1 (0.39) | 0.07 |
| 1×10^5^ | 28.0 (0.27) | 27.3 (0.15) | 0.40 |
| 1×10^4^ | 30.6 (0.40) | 30.8 (0.18) | >0.99 |
| 1×10^3^ | 34.0 (0.43) | 33.9 (0.84) | >0.99 |
| 1×10^2^ | 36.3 (0.58) | 36.0 (0.77) | >0.99 |
| 1×10^1^ | 36.9 (0.92) | 36.6 (0.73) | 0.98 |
