# Supplementary material for: Optimization and prospective evaluation of sensitive real-time PCR assays with an internal control for the diagnosis of melioidosis in Thailand
Source: Microbiol Spectr. 2023 Oct 11;11(6):e01039-23. doi: 10.1128/spectrum.01039-23 (PMC10715024; doi:10.1128/spectrum.01039-23)
Supplement: Table S6 — Real-time PCR results of individual samples from patients (N = 87). [file spectrum.01039-23-s0007.docx]

**Table S6:** Real-time PCR results of individual samples from patients (N = 87)

| **Sample code** | **Type of patients** | **TTS1-*orf2* Ct value** | **BPSS0745  Ct value** | **BPSS1187  Ct value** | **BPSS1498 Ct value** |
| --- | --- | --- | --- | --- | --- |
| ICT-MdH 003 | Bacteremic melioidosis | 33.62 | 31.64 | 32.23 | 30.88 |
| ICT-MdH 020 | Bacteremic melioidosis | Not detected | Not detected | Not detected | Not detected |
| ICT-MdH 027 | Bacteremic melioidosis | Not detected | 36.92 | 36.43 | Not detected |
| ICT-MdH 031 | Bacteremic melioidosis | 31.85 | 32.18 | 32.06 | 31.53 |
| ICT-MdH 045 | Bacteremic melioidosis | 25.84 | 26.96 | 27.15 | 26.29 |
| ICT-MdH 048 | Bacteremic melioidosis | Not detected | 36.77 | Not detected | Not detected |
| ICT-MdH 056 | Bacteremic melioidosis | 36.91 | 32.31 | 32.55 | 30.98 |
| ICT-MdH 060 | Bacteremic melioidosis | Not detected | Not detected | Not detected | Not detected |
| ICT-MdH 061 | Bacteremic melioidosis | Not detected | Not detected | 36.51 | 33.76 |
| ICT-MdH 066 | Bacteremic melioidosis | 34.63 | 31.97 | 32.71 | 30.79 |
| ICT-MdH 070 | Bacteremic melioidosis | Not detected | 37.2 | 37.02 | 35.96 |
| ICT-MdH 072 | Bacteremic melioidosis | 34.9 | 32.17 | 32.55 | 30.37 |
| ICT-MdH 075 | Bacteremic melioidosis | Not detected | 37.21 | 35.54 | 34 |
| ICT-MdH 077 | Bacteremic melioidosis | Not detected | 33.44 | 33.63 | 34.56 |
| ICT-MdH 078 | Bacteremic melioidosis | Not detected | Not detected | 37.5 | Not detected |
| ICT-MdH 079 | Bacteremic melioidosis | 38.09 | 32.57 | 33.2 | 31.83 |
| ICT-MdH 081 | Bacteremic melioidosis | Not detected | Not detected | 39.2 | Not detected |
| ICT-MdH 083 | Bacteremic melioidosis | 43.16 | 33.53 | 33.43 | 31.86 |
| ICT-MdH 087 | Bacteremic melioidosis | Not detected | 35.89 | 36.99 | 44.48 |
| ICT-MdH 089 | Bacteremic melioidosis | 28.05 | 27.95 | 27.97 | 26.3 |
| ICT-MdH 090 | Bacteremic melioidosis | Not detected | 37.14 | Not detected | 35.24 |
| ICT-MdH 092 | Bacteremic melioidosis | Not detected | 35.38 | 37.08 | Not detected |
| ICT-MdH 095 | Bacteremic melioidosis | 31.59 | 29.64 | 29.8 | 28.49 |
| ICT-MdH 097 | Bacteremic melioidosis | 43.23 | 34.73 | 33.56 | 31.89 |
| ICT-MdH 103 | Bacteremic melioidosis | 43.93 | 34.46 | 34.29 | 34.31 |
| ICT-MdH 111 | Bacteremic melioidosis | Not detected | 31.7 | 31.55 | 31.36 |
| ICT-MdH 114 | Bacteremic melioidosis | Not detected | Not detected | 37.66 | Not detected |
| ICT-MdH 115 | Bacteremic melioidosis | Not detected | 32.63 | 32.79 | 32.55 |
| ICT-MdH 119 | Bacteremic melioidosis | 34.02 | 31.69 | 31.52 | 30.11 |
| ICT-MdH 132 | Bacteremic melioidosis | Not detected | 37.36 | 35.74 | 34.16 |
| ICT-MdH 134 | Bacteremic melioidosis | 33.51 | 30.98 | 31.5 | 29.82 |
| ICT-MdH 138 | Bacteremic melioidosis | Not detected | Not detected | Not detected | Not detected |
| ICT-MdH 146 | Bacteremic melioidosis | Not detected | 35.54 | 38.69 | Not detected |
| ICT-MdH 156 | Bacteremic melioidosis | 26.58 | 27.39 | 27.81 | 25.89 |
| ICT-MdH 159 | Bacteremic melioidosis | Not detected | Not detected | Not detected | Not detected |
| ICT-MdH 160 | Bacteremic melioidosis | Not detected | 35.1 | 34.66 | 33.74 |
| ICT-MdH 167 | Bacteremic melioidosis | Not detected | Not detected | Not detected | Not detected |
| ICT-MdH 172 | Bacteremic melioidosis | 38.15 | 33.08 | 33 | 32.81 |
| ICT-MdH 004 | Non-melioidosis | Not detected | Not detected | Not detected | Not detected |
| ICT-MdH 005 | Non-melioidosis | Not detected | Not detected | Not detected | Not detected |
| ICT-MdH 008 | Non-melioidosis | Not detected | Not detected | Not detected | Not detected |
| ICT-MdH 011 | Non-melioidosis | Not detected | Not detected | Not detected | Not detected |
| ICT-MdH 012 | Non-melioidosis | Not detected | Not detected | Not detected | Not detected |
| ICT-MdH 014 | Non-melioidosis | Not detected | Not detected | Not detected | Not detected |
| ICT-MdH 018 | Non-melioidosis | Not detected | Not detected | Not detected | Not detected |
| ICT-MdH 021 | Non-melioidosis | Not detected | Not detected | Not detected | Not detected |
| ICT-MdH 025 | Non-melioidosis | Not detected | Not detected | Not detected | Not detected |
| ICT-MdH 026 | Non-melioidosis | Not detected | Not detected | Not detected | Not detected |
| ICT-MdH 028 | Non-melioidosis | Not detected | Not detected | Not detected | Not detected |
| ICT-MdH 032 | Non-melioidosis | Not detected | Not detected | Not detected | Not detected |
| ICT-MdH 035 | Non-melioidosis | Not detected | Not detected | Not detected | Not detected |
| ICT-MdH 037 | Non-melioidosis | Not detected | Not detected | Not detected | Not detected |
| ICT-MdH 038 | Non-melioidosis | Not detected | Not detected | Not detected | Not detected |
| ICT-MdH 042 | Non-melioidosis | Not detected | Not detected | Not detected | Not detected |
| ICT-MdH 043 | Non-melioidosis | Not detected | Not detected | Not detected | Not detected |
| ICT-MdH 044 | Non-melioidosis | Not detected | Not detected | Not detected | Not detected |
| ICT-MdH 046 | Non-melioidosis | Not detected | Not detected | Not detected | Not detected |
| ICT-MdH 050 | Non-melioidosis | Not detected | Not detected | Not detected | Not detected |
| ICT-MdH 051 | Non-melioidosis | Not detected | Not detected | Not detected | Not detected |
| ICT-MdH 053 | Non-melioidosis | Not detected | Not detected | Not detected | Not detected |
| ICT-MdH 054 | Non-melioidosis | Not detected | Not detected | Not detected | Not detected |
| ICT-MdH 055 | Non-melioidosis | Not detected | Not detected | Not detected | Not detected |
| ICT-MdH 059 | Non-melioidosis | Not detected | Not detected | Not detected | Not detected |
| ICT-MdH 063 | Non-melioidosis | Not detected | Not detected | Not detected | Not detected |
| ICT-MdH 064 | Non-melioidosis | Not detected | Not detected | Not detected | Not detected |
| ICT-MdH 067 | Non-melioidosis | Not detected | Not detected | Not detected | Not detected |
| ICT-MdH 080 | Non-melioidosis | Not detected | Not detected | Not detected | Not detected |
| ICT-MdH 084 | Non-melioidosis | Not detected | Not detected | Not detected | Not detected |
| ICT-MdH 091 | Non-melioidosis | Not detected | Not detected | Not detected | Not detected |
| ICT-MdH 093 | Non-melioidosis | Not detected | Not detected | Not detected | Not detected |
| ICT-MdH 099 | Non-melioidosis | Not detected | Not detected | Not detected | Not detected |
| ICT-MdH 100 | Non-melioidosis | Not detected | Not detected | Not detected | Not detected |
| ICT-MdH 110 | Non-melioidosis | Not detected | Not detected | Not detected | Not detected |
| ICT-MdH 113 | Non-melioidosis | Not detected | Not detected | Not detected | Not detected |
| ICT-MdH 124 | Non-melioidosis | Not detected | Not detected | Not detected | Not detected |
| ICT-MdH 127 | Non-melioidosis | Not detected | Not detected | Not detected | Not detected |
| ICT-MdH 129 | Non-melioidosis | Not detected | Not detected | Not detected | Not detected |
| ICT-MdH 141 | Non-melioidosis | Not detected | Not detected | Not detected | Not detected |
| ICT-MdH 142 | Non-melioidosis | Not detected | Not detected | Not detected | Not detected |
| ICT-MdH 144 | Non-melioidosis | Not detected | Not detected | Not detected | Not detected |
| ICT-MdH 147 | Non-melioidosis | Not detected | Not detected | Not detected | Not detected |
| ICT-MdH 153 | Non-melioidosis | Not detected | Not detected | Not detected | Not detected |
| ICT-MdH 157 | Non-melioidosis | Not detected | Not detected | Not detected | Not detected |
| ICT-MdH 164 | Non-melioidosis | Not detected | Not detected | Not detected | Not detected |
| ICT-MdH 165 | Non-melioidosis | Not detected | Not detected | Not detected | Not detected |
| ICT-MdH 170 | Non-melioidosis | Not detected | Not detected | Not detected | Not detected |
| ICT-MdH 174 | Non-melioidosis | Not detected | Not detected | Not detected | Not detected |
