# Supplementary material for: Optimization and prospective evaluation of sensitive real-time PCR assays with an internal control for the diagnosis of melioidosis in Thailand
Source: Microbiol Spectr. 2023 Oct 11;11(6):e01039-23. doi: 10.1128/spectrum.01039-23 (PMC10715024; doi:10.1128/spectrum.01039-23)
Supplement: Table S7 — Organisms isolated from clinical samples of 99 other infection patients. [file spectrum.01039-23-s0008.docx]

**Table S7:** Organisms isolated from clinical samples of 99 other-infection patients

| **Infectious agents** | **Total number of samples** |
| --- | --- |
| **Bacteria** |  |
| *Acinetobacter baumannii* | 11 |
| Alpha-hemolytic Streptococci | 2 |
| *Bacillus* spp. | 3 |
| Beta-hemolytic Streptococci | 1 |
| *Corynebacterium* spp. | 1 |
| *Enterobacter cloacae* | 1 |
| *Enterococcus faecalis* | 12 |
| *Enterococcus faecium* | 1 |
| *Escherichia coli* | 15 |
| Group D *Streptococcus* | 3 |
| *Klebsiella oxytoca* | 1 |
| *Klebsiella penumoniae* | 14 |
| *Pseudomonas aeruginosa* | 2 |
| *Salmonella* serogroup D | 1 |
| *Salmonella* spp. | 1 |
| *Staphylococcus aureus* | 5 |
| *Staphylococcus haemolyticus* | 2 |
| *Staphylococcus saprophyticus* | 1 |
| *Streptococcus agalactiae* | 1 |
| *Streptococcus pyogenes* | 4 |
| *Vibrio parahaemolyticus* | 1 |
| *Vibrio* spp. | 1 |
| Mixed infections | 15 |
| **Total** | 99 |
