# Supplementary material for: Optimization and prospective evaluation of sensitive real-time PCR assays with an internal control for the diagnosis of melioidosis in Thailand
Source: Microbiol Spectr. 2023 Oct 11;11(6):e01039-23. doi: 10.1128/spectrum.01039-23 (PMC10715024; doi:10.1128/spectrum.01039-23)
Supplement: Table S8 — Comparison of the BPSS1187 real-time PCR assay (N = 19) between the CFX96 Touch Real-Time PCR system (Bio-Rad) and the Quantstudio 1 Real-Time PCR system (Applied Biosystems) with FAM and HEX fluorescence signals. [file spectrum.01039-23-s0009.docx]

**Table S8:** Comparison of BPSS1187 real-time PCR assay (N = 19) between CFX96 Touch Real-Time PCR system (Bio-Rad) and Quantstudio 1 Real-Time PCR system (Applied Biosystems) with FAM and HEX fluorescence signals.

| **Type of samples** | **Sample code** | **FAM** | | **HEX** | |
| --- | --- | --- | --- | --- | --- |
|  |  | **CFX96** | **Quantstudio 1** | **CFX96** | **Quantstudio 1** |
| Target detected | iTP-MdH-024 Synovial | 18.39 | 18.94 | Not detected | Not detected |
|  | BP_K96243 | 32.37 | 32.57 | Not detected | Not detected |
|  | iTP-MdH-019 Plasma | 34.04 | 34.4 | 34.29 | 34.06 |
|  | iTP-MdH-012 Plasma | 34.31 | 33.06 | 32.87 | 33.24 |
|  | iTP-MdH-107 Plasma | 35.35 | 36.32 | 36.11 | 36.29 |
|  | iTP-MdH-026 Plasma | 35.46 | 35.24 | 31.9 | 32.83 |
|  | iTP-MdH-201 Plasma | 35.6 | 36.06 | Not detected | Not detected |
|  | iTP-MdH-171 Urine | 36.83 | 34.81 | 30.08 | 29.33 |
|  | 150 D0 Urine | 39.46 | 37.22 | N/A | N/A |
|  | BC 3 | 41.4 | 40.45 | 25.98 | 25.4 |
| No target detected | 68PM D4 | Not detected | Not detected | N/A | N/A |
|  | Healthy 01 | Not detected | Not detected | N/A | N/A |
|  | Healthy 02 | Not detected | Not detected | N/A | N/A |
|  | BC 27 | Not detected | Not detected | 26.28 | 26.16 |
|  | BC 31 | Not detected | Not detected | 25.24 | 25.15 |
|  | BC 56 | Not detected | Not detected | 27.2 | 26.92 |
|  | BC 70 | Not detected | Not detected | 26.68 | 26.55 |
|  | BC 75 | Not detected | Not detected | 26.16 | 25.75 |
|  | iTP-MdH-084 Urine | Not detected | Not detected | Not detected | Not detected |

N/A = No target in sample.

Not detected = Thermocycler cannot detect the target.

CFX96 = CFX96 Touch Real-Time PCR System (Bio-Rad, Hercules, CA)

Quantstudio1 = Quantstudio 1 Real-Time PCR system (Applied Biosystems, USA)
