# Supplementary material for: Optimization and prospective evaluation of sensitive real-time PCR assays with an internal control for the diagnosis of melioidosis in Thailand
Source: Microbiol Spectr. 2023 Oct 11;11(6):e01039-23. doi: 10.1128/spectrum.01039-23 (PMC10715024; doi:10.1128/spectrum.01039-23)
Supplement: Table S9 — Real-time PCRs and culture results of clinical samples from melioidosis patients (N = 140). [file spectrum.01039-23-s0010.docx]

**Table S9:** Real-time PCRs and culture results of clinical samples from melioidosis patients (N = 140)

| **Clinical samples** | **Mukdahan hospital (%)** | | | | **Roi Et hospital (%)** | | | | **Total (%)** | | | |
| --- | --- | --- | --- | --- | --- | --- | --- | --- | --- | --- | --- | --- |
|  | **Total** | **TTS1-*orf2*** | **BPSS1187** | **Culture** | **Total** | **TTS1-*orf2*** | **BPSS1187** | **Culture** | **Total** | **TTS1-*orf2*** | **BPSS1187** | **Culture** |
| Plasma | 30 | 19 (63.3) | 29  (96.7) | 24  (80) | 44 | 29 (65.9) | 34  (77.3) | 41 (93.2) | 74 | 48 (64.9) | 63  (85.1) | 65 (87.8) |
| Pus | 4 | 4  (100) | 4  (100) | 4  (100) | 2 | 1  (50) | 2  (100) | 2  (100) | 6 | 5  (83.3) | 6  (100) | 6  (100) |
| Sputum | 7 | 6  (85.7) | 6  (85.7) | 6 (85.7) | 16 | 13 (81.3) | 13  (81.3) | 10 (62.5) | 23 | 19 (82.6) | 19  (82.6) | 16 (69.6) |
| Body fluid | 1 | 0  (0) | 1  (100) | 1  (100) | 1 | 1  (100) | 1  (100) | 1  (100) | 2 | 1  (50) | 2  (100) | 2  (100) |
| Urine | 10 | 4  (40) | 4  (40) | 2  (20) | 25 | 14  (56) | 17  (68) | 3  (12) | 35 | 18 (51.4) | 21  (60) | 5  (14.3) |
